# New Advances in the Phenolic Composition of Tiger Nut (*Cyperus esculentus* L.) by-Products

**DOI:** 10.3390/foods11030343

**Published:** 2022-01-25

**Authors:** María del Carmen Razola-Díaz, Ana María Gómez-Caravaca, Eduardo J. Guerra-Hernández, Belén Garcia-Villanova, Vito Verardo

**Affiliations:** 1 Department of Nutrition and Food Science, Campus of Cartuja, University of Granada, 18071 Granada, Spain; carmenrazola@ugr.es (M.d.C.R.-D.); ejguerra@ugr.es (E.J.G.-H.); belenv@ugr.es (B.G.-V.); 2 Institute of Nutrition and Food Technology ‘José Mataix’, Biomedical Research Centre, University of Granada, Avd. Conocimiento s/n, 18100 Granada, Spain; anagomez@ugr.es; 3 Department of Analytical Chemistry, Faculty of Sciences, University of Granada, Avd. Fuentenueva s/n, 18071 Granada, Spain

**Keywords:** ultrasound-assisted extraction, phenolic compounds, antioxidant activity, chufa, HPLC-ESI-TOF-MS

## Abstract

“Horchata” is a well-known Spanish beverage obtained from pressing tiger nuts. Its by-product is a potential source of sugar and fiber but also contains polyphenols; thus, it could be used as a new ingredient in the food industry. The aim of this work is to determine the phenolic compounds and compare the phenolic profile of two tiger nut by-products. A Box–Behnken design has been carried out to optimize the extraction of phenolic compounds from tiger nut by-products by ultrasound technology. The independent factors were time (min), ethanol/water (% *v/v*), and solvent/sample ratio (*v/w*). The model was validated and confirmed by ANOVA. A Protected Designation of Origin (PDO) of Valencia and a non-Protected Designation of Origin (n-PDO) tiger nut by-products were extracted under the optimal conditions and were characterized by HPLC-DAD-ESI-TOF-MS (High Performance Liquid Chropatography coupled to a photodiode array time-of-flight mass detector). Moreover, their antioxidant activities measured by three different methods (DPPH (2,2-diphenyl-1-picrylhydrazyl), ABTS (2,2′-Azinobis [3-ethylbenzothiazoline-6-sulfonic acid]-diammonium salt) and FRAP (ferric reducing antioxidant power)) were compared. A total of 45 polar compounds were identified, and the phenolic ones were quantified, some of them for the first time. PDO tiger nut by-product has been demonstrated to be richer in phenolic acids and other polyphenols and has higher antioxidant activity; meanwhile, n-PDO tiger nut by-product is richer in phenol precursors.

## 1. Introduction

Tiger nut (*Cyperus esculentus* L.) is a tuber mainly used to obtain a tiger nut beverage with a milky appearance, called “horchata”. It is typically from Spain, where the horchata production industry supposes €60 million per year. The by-products generated from this industry are up to 60% of the tiger nut used, which equals 1.8 million kg per year. Tiger nut by-products are mainly used for animal feed or as organic matter for combustion [1], as a carbon source for the growth of probiotic bacteria [2], or as a sugar source for microalgae [3]. Besides, there are several studies that have evaluated the potential of this by-product as a source of fiber to enrich meat products such as pork [4], beef [5] burgers, and pork sausages [6], to enhance cooking performance, moisture and fat retention. However, there are only a few studies concerning the potential use of this by-product for food and nutraceutical industries. Some studies have evaluated enzyme pretreatments combined with high pressure [7] or different mixtures of solvents at different temperatures [8] to extract phenolic compounds from tiger nut by-product. Nevertheless, they only used spectrophotometric methods to quantify total phenolic compounds, but individual components were not quantified nor chemically characterized. More recent studies have characterized the phenolic profile of the tiger nut by-product extracted by conventional extraction [9] or of the tiger nut by-product oil by supercritical fluid extraction with CO_2_ [10]. Clemente-Villalba et al. (2021) [11] have recently published a comparison of the sensory profile, volatile composition, and consumer’s acceptance of PDO and n-PDO tiger nut milks. Razola Díaz et al. (2020) [3] compared the chemical composition and sugar content in PDO and n-PDO tiger nut by-products. Thus, the objective of the present study is to compare, for the first time, the phenolic profiles of two tiger nut by-products from different origins (PDO and n-PDO) that were obtained during production at an industrial scale. The extraction of phenolic compounds was optimized using ultrasound technology with a Box–Behnken design. All the polar compounds were tentatively identified by HPLC-ESI-TOF-MS, and the phenolics were quantified. The antioxidant activity of both by-products was measured by three different methods (DPPH, ABTS, and FRAP).

## 2. Materials and Methods

### 2.1. Chemicals and Samples

HPLC (High Performance Liquid Chromatography)-grade water and other reagents and solvents were purchased from Merck KGaA (Darmstadt, Germany).

Tiger nut by-product samples were provided by Puleva company located in Granada, Spain, in May 2019. Two types of samples were collected, one from Protected Designation of Origin (PDO) of Valencia and another one without appellation of origin (non-Protected Designation of Origin (n-PDO)) from Ivory Coast. The PDO by-product was obtained after three successive pressings of the tiger nut, while the n-PDO by-product was obtained after only two presses due to its original composition and the final target product of the company as the non-PDO tiger nut was richer in fat. The samples were dried in a ventilated oven at 40 °C until obtaining 5.57 ± 0.29 and 5.46 ± 0.67 % of humidity in the PDO and n-PDO tiger nut by-product, respectively, and milled and sieved to 100 µm; after that, they were frozen at −18 °C until the analyses.

### 2.2. Experimental Design

A Box–Behnken design composed of 15 experiments in three levels (−1,0,1) was established to optimize the extraction of phenolic compounds from tiger nut by-products. All the parameters of the model were established according to the previous experience of the group and previous trials. The independent variables were time (5, 45 and 85 min), ratio ethanol/water (0, 50 and 100% *v/v*), and ratio solvent/sample (20, 40 and 100 *v/w*), and the response was the total phenolic content measured using the Folin–Ciocalteu method. The model was fitted to a second-order polynomial equation, and its adjustment was evaluated and confirmed by ANOVA. To select the optimal conditions, response surface methodology (RSM) was used. All the data was processed using STATISTICA 7.0 (2002, StatSoft, Tulsa, OK, USA).

### 2.3. Ultrasound Bath Extraction

Briefly, 0.125 g of tiger nut by-product powder was dissolved in a 10 mL solution of ethanol/water 40/60, *v/v*. The mixture was placed in an ultrasonic bath for 50 min, and then it was centrifuged for 10 min at 9000 rpm. The supernatant was collected, evaporated, and reconstituted in 1 mL of methanol/water (50:50, *v/v*). The final extracts were filtered with regenerated cellulose filters 0.2 μm (Millipore, Bedford, MA, USA) and stored at −18 °C until the analyses.

### 2.4. Determination of Total Phenolic Content (TPC)

Folin–Ciocalteu spectrophotometric method was used to determine the TPC in tiger nut by-product [12]. Thus, 100 µL of the extract was added to 500 µL of the Folin–Ciocalteu reagents and 6 mL of bi-distilled water. The flask was agitated for a minute. After that, 2 mL of 15% (*w/v*) Na_2_CO_3_ and made up to 10 mL with bi-distilled water. The flasks were kept in darkness. After 2 h, the measures were carried out at 750 nm and 25 °C with a UV-visible spectrophotometer (Spectrophotometer 300 Array, UV-Vis, single beam, Shimadzu, Duisburg, Germany). The calibration curve was developed with Gallic acid from 1 to 100 ppm, and the obtained equation was y = 0.0012x + 0.0227 (R^2^ = 0.9995). Results are expressed as µg gallic acid equivalents (GAE)/g dry weight (d.w.).

### 2.5. Determination of Polar Compounds by HPLC-ESI-QTOF-MS

The phenolic profile characterization and quantification of the two tiger nut by-products extracted by the optimal conditions were performed according to a previously described method [13]. The analyses were carried out in duplicate on an ACQUITY Ultra Performance LC system (Waters Corporation, Milford, MA, USA) coupled to an electrospray ionization (ESI) source operating in the negative mode and a time-of-flight (TOF) mass detector (Waters Corporation, Milford, MA, USA). The compounds of interest were separated on an ACQUITY UPLC BEH Shield RP18 column (1.7 µm, 2.1 mm × 100 mm; Waters Corporation, Milford, MA, USA) at 40 °C using a gradient previously stated by Verni et al. [13] using water containing 1% acetic acid as mobile phase A and acetonitrile as mobile phase B. The gradient was: from 0 to 2.3 min, 1% B; 4.4 min, 7% B; 8.1 min, 14% B; 12.2 min, 24% B; 16 min, 40% B; 18.3 min, 100% B, 21 min, 100% B; 22.4 min, 1% B; 25 min, 1% B. Flow rate was established to 0.6 mL/min. The volume injection was 2 µL.

Finally, external calibration curves were prepared for the quantification of phenolic compounds: vanillic acid (y = 99.369x + 40.864, R^2^ = 0.9995), ferulic acid (y = 173.03x + 64.326, R^2^ = 0.9958), rutin (y = 1137.8x + 74.815, R^2^ = 0.9943), quercetin (y = 1272.5x + 84.49, R^2^ = 0.9938), and catechin (y = 317.69x + 105.65, R^2^ = 0.9955). The data were elaborated using MassLynx 4.1 software (Waters Corporation, Milford, MA, USA).

### 2.6. Antioxidant Assays in Tiger Nut By-Products

The antioxidant capacity in the two tiger nut by-products has been evaluated in the extract obtained by the optimal ultrasound bath conditions using three different methods.

The ABTS method was carried out according to Re et al. (1999) [14]. The monocation ABTS^•+^ is generated by oxidation of the ABTS with potassium persulfate in the dark at room temperature for 12–24 h. For each extract, 1 mL of this ABTS solution was added to 0.01 mL of the extract and the detriment of absorbance during 30 min at 734 nm was measured.

The DPPH radical scavenging activity was assayed with a method proposed by several authors [15,16]. In total, 100 µL of each extract was added to 2.9 mL of DPPH, and after rapid stirring, the bleaching power of the extract was observed in a time interval from 0 to 30 min at 517 nm.

The FRAP assay was carried out following the procedure developed by Pulido et al. (2000) [17]. It is based on the reduction of Fe^3+^ to Fe^2+^ by the antioxidant substances. A total of 30 µL of each extract was added to 90 µL of distilled water and 900 µL of the FRAP reagent. It was kept for 30 min at 37 °C and measured in the spectrophotometer at 595 nm.

Standard curves of Trolox equivalents (TE) (1, 5, 10, 20, 50, 80, 100, 150, 200 ppm) were elaborated for each assay and the equations obtained were y = 0.0009x + 0.0258 (R^2^ = 0.9971), y = 0.0026x + 0.0374 (R^2^ = 0.9963), and y = 0.0039x + 0.0915 (R^2^ = 0.9972), for the ABTS, DPPH, and FRAP assays, respectively. Results are expressed as mg TE/g d.w.

## 3. Results and Discussion

### 3.1. Fitting the Model

The extraction step is the most important to reach the highest amount of the target compounds, in this case, phenolic compounds. A Box–Behnken design coupled to RSM was used to find the optimal conditions of time (X_1_), ethanol/water ratio (X_2_), and solvent/sample ratio (X_3_) to extract phenolic compounds from the tiger nut by-product using ultrasound bath technology. The experimental values of TPC obtained for each run are shown in Table 1. The lowest recovery (42.80 ± 1.08 µg GAE/g d.w.) was at 45 min, with the ethanol/water 100% and ratio solvent/sample 20 *v/w*, and the highest (383.11 ± 1.76 µg GAE/g d.w.) at 85 min, was ethanol/water 50% and ratio solvent/sample 100 *v/w*. This by-product has a low relation weight/volume (<1); therefore, those experiments carried out with the lower value of ratio solvent/sample led to the lowest results due to the saturation of the solvent and consequently the reduced contact surface between sample and solvent. Similar results were found when the tendency using ethanol was 100%; therefore, it demonstrates that some water is needed as surfactant for the extraction of phenolic compounds to satisfactorily take place.

The experimental data were adjusted to a second-order polynomial equation, and all the estimated regression effects are shown in Table 2. The model was analyzed with a significance level of *p* < 0.05, and all the lineal terms (β_1_, β_2_, and β_3_), all the quadratic terms (β_11_, β_22_ and β_33_), and the crossed values between time (X_1_) and ratio solvent/sample (X_3_), had significance, but the rest of the terms were discarded. Therefore, the model was recalculated, and the ANOVA test was performed. As shown in Table 2 the model revealed a high correlation coefficient (R^2^ = 0.9891), a significant regression model (*p* < 0.05), and a non-significant lack of fit (*p* > 0.05). According to Bezerra et al. [18], the adequacy of the model was confirmed.

**Figure 1:**
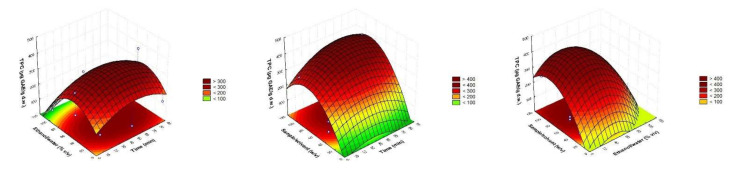
Response surface plots showing combined effects of process variables.

Optimal conditions were selected using RSM concerning the three-dimensional graphs presented in Figure 1. A compromise has been made between the independent factors to establish them at the minimal possible values. The optimal conditions chosen were: 50 min, 40% ethanol/water, and ratio solvent/sample 80 *v/w* that gave a predicted value of 403.61 ± 49.54 µg GAE/g d.w. The coefficient of variation between the obtained (400.43 ± 4.63) and predicted values was lower than 1%, so the model was validated.

The optimal result obtained was better than the one reported by Roselló-Soto et al. [8] that used conventional extraction with a similar solvent (ethanol 25%) but with differences in terms of time (3 h) and temperature (60 °C). Therefore, ultrasound technology has been demonstrated to be a non-thermal and lower time-consuming technique that allows the satisfactorily recovery of phenolic compounds from tiger nut by-products. However, the authors reported slightly higher results using 50% ethanol, 35 °C, and pH 2.5 for 3 h [9]. In our case, the pH has not been changed in order to avoid the hydrolysis of bound compounds.

### 3.2. Identification of Polar Compounds by HPLC-ESI-TOF-MS

The samples of PDO and n-PDO tiger nut by-products were characterized by HPLC-MS, and 44 compounds were identified, 18 were phenolic compounds and 26 other polar compounds were identified as hydroxyl fatty acids. To our knowledge, a total of 27 polar compounds were identified for the first time in tiger nut by-products.

All the identified compounds are described in Table 3 with their retention time, molecular formula, experimental and calculated *m/z* fragments, and score and error (ppm). To ensure mass accuracy, the tolerances chosen had a score higher than 90% and an error lower than 5 ppm (part per million) between the experimental and calculated *m/z*. To identify the compounds, the generated molecular formula and some in source fragments were checked and studied also comparing with different databases such as PubChem, Phenol-Explorer, and the literature.

Five phenolic acids were identified at times 1.34, 2.57, 3.88, 5.51, and 5.73 min (peaks 4, 6, 7, 9, and 19). They were named *p*-hydroxybenzoic acid, vanillic acid, ethyl vanillin, ferulic acid, and *p*-coumaric acid, respectively. In addition, peaks 2, 8, 15, and 21 were assigned to scopoletin (a coumarin), 4-vinylphenol (a hydroxy styrene), sinensetin (a methylated flavone), and cyanidin (an anthocyanidin), with *m/z* 191, 119, 371, and 286, respectively. These compounds are in agreement with Roselló-Soto et al. (2019) [9,10] who previously described them in tiger nut by-product. As they described some vanillin and *p*-coumaric acid derivates, the compounds found at 9.47 and 12.67 min (peaks 13 and 17) were named dehydrovanillin and *p*-coumaric acid ethyl ester, respectively. Overall vanillic and *p*-coumaric acid derivates described here were attributed to have good bioavailability and bio-accessibility and to reduce the risk of developing several disorders such as cancer and cardiovascular diseases [19].

At time 8.25 min (peak 8), the compound kaempferol 3,7-diglucoside has been identified according to its mass fragments 285 and 447. This compound could also be named sophoraflavonoloside or luteolin-7,3′-di-*O*-glucoside. Peak 1 had been previously detected but not quantified in fruits. It was named 2-*O*-galloyl-1,4-galactarolactone [20]. With the fragment *m/z* 85 corresponding with peak 5, l-leucic acid was tentatively identified. At time 1.16 (peak 3), with molecular formula C_26_H_20_O_7_ and the fragment with *m/z* 229, imbricantonol was found according to Byrne et al. (1987) [21], who found this compound for the first time in *Stypandra glauca* (blind grass). According to the authors, the fragments found for this compound indicate the possible couple of dianellidin (*m/z* 215) with stypandrone (*m/z* 229) and the presence of three hydroxyl substituents (*m/z* 570). Peak 11 was identified as aspalathin (3-hydroxyphloretin 2′-*O*-glucoside), previously described as the major compound in *Aspalathus linearis* (rooibos) by Kreuz et al. (2008) [22]. At *m/z* 683 (peak 14) and fragments at *m/z* 721, 593 and 563, the veronicafolin 3-glucosyl-(1->3)-galactoside has been tentatively identified according to Tan et al. (2020) [23] and previously described in *Clinacanthus nutans*, a popular herbal plant in the Southeast Asian region. Previously some studies have found flavonoids as kaempferol glycosides to have several roles in human health, including antioxidant, anti-inflammatory, antimicrobial, antidiabetic, anticancer, anti-osteoporotic, cardioprotective, anxiolytic, analgesic, and neuroprotective, among others [24].

Sinapyl alcohol and benzoic acid were identified at 11.64 and 16.13 min, corresponding with peaks 16 and 22. Sinapyl alcogol has been described as a precursor to various stilbenes and coumarins, and benzoic acid as a precursor of phenolic acids [25].

The compounds with molecular formula C_18_H_34_O_5_ and fragments at *m/z* 229, 211 and 171 (peaks 18, 19 and 20) were identified as trihydroxy octadecenoic acid isomers. They also could be named pinellic acid isomers, according to Ruan et al. (2019), who described them in *Pluchea indica* aerial parts [26]. Peaks 37–40 and 42–45 were identified as fatty acids, linolenic, myristic, palmitoleic, linoleic, palmitic, oleic, heptadecanoic, and stearic, according to several authors that found them in tiger nut oil [27,28,29]. Hydroxyoleic acid isomers (peaks 31–33, 35) and dihydroxyoleic acids isomers (peaks 23–26), derived from oleic acid, have also been found. Other derivates were found at different times: Hydroxystearic and dihydroxystearic acids at 17.54 and 16.98 min, hydroxylinoleic acid isomers at 17.09, 17.20, and 17.44 min, and hydroxypalmitic and methylpalmitic acids at 17.12 and 18.00 min. Another fatty acid tentatively identified was trihydroxy octadecenoic acid isomers at peaks 18–20. None of which were previously described in tiger nut by-products.

### 3.3. Quantification of Phenolic Compounds by HPLC-ESI-TOF-MS and Antioxidant Activity

A total of 18 phenolic compounds were quantified, and they are summarized in Table 4.

Comparing the phenolic compounds (Table 4) in both tiger nut by-products, the PDO sample clearly has a higher amount of 2-*O*-galloyl-1,4-galactarolactone, scopoletin, imbricantonol, p-hydroxybenzoic acid, vanillic acid, ethyl vanillin, 4-vinylphenol, ferulic acid, *p*-coumaric acid, and *p*-coumaric acid ethyl ester. On the other hand, n-PDO by-product is sightly richer in cyanidin, veronicafolin 3-glucosyl-(1->3)-galactoside, and the two phenolic compounds precursors, benzoic acid and sinapyl alcohol. Similar amounts of 3-hydroxyphloretin 2′-*O*-glucoside (aspalathin), kaempferol 3,7-diglucoside, and sinensetin were found in both samples.

The phenolic composition, mainly based on phenolic acids, justified the optimal conditions of extraction obtained in this work. In fact, according to Waszkowiak and Gliszczynska-Swigło, the phenolic acid content decreases when ethanol concentration is higher than 60 to 70 % [30]. Similar data were reported by Roselló-Soto et al. [9], obtaining the highest recovery of phenolic acids in tiger nuts using 41.4 % ethanol.

Compared with other authors, we have achieved a higher amount of cyanidin, 4-vinylphenol, ethyl vanillin, and p-coumaric acid than Roselló-Soto et al. [9]. This could be because they used a conventional extraction while we used ultrasound bath assisted extraction; however, the difference between the samples must also be considered. No other references were found in the bibliography regarding phenolic compounds in tiger nut by-products.

In addition, PDO and n-PDO tiger nut by-products were revealed to have antioxidant activity (Table 5), being around 15% higher for the PDO by-products with the three performed methods. Antioxidant activity has been reported previously in tiger nut oils [31] and tiger nut beverages [32]. However, no references were found in tiger nut by-products apart from those reported by Roselló-Soto et al. (2019) [9], but the method used was different (total antioxidant capacity).

Roselló-Soto et al. (2018) [33] reported a total of 30 phenolic compounds that were previously found and quantified in tiger nut tubers. Parts of these phenols naturally remian in the horchata after the pressing steps; therefore, in the by-product, fewer compounds remain and in lower amounts. This corroborates the results obtained in this work as 18 phenolic compounds were identified and quantified in tiger nut industrial by-products. Besides, tiger nut by-products have a lower amount of phenolic compounds with lower antioxidant activity than tiger nut oils [31,34] and tiger nut by-product oils [10]. This is mainly because of the oil extraction techniques that hydrolysate the compounds. However, the use of ultrasound technology seems to be a promising technique to apply to tiger nut by-products that allow obtaining phenolic compounds with antioxidant activity. This makes it clear that the PDO tiger nut by-product contains higher amounts of phenolic compounds than the n-PDO tiger nut by-product. This result was realized as tiger nuts cultivated in Valencia (Spain) contain higher amounts of these antioxidants compared to the samples from Ivory Coast that contain more precursors. This difference could be justified with the growth differentiation balance framework. According to this hypothesis, when there are high levels of nitrogen and good environmental conditions, the growth of the plant is favored. On the contrary, if the environmental conditions are not favorable and the availability of essential components is low, the secondary metabolism is favored increasing the phenolic content. However, to confirm this trend, further analyses on the most robust sampling are needed.

## 4. Conclusions

The ultrasound-assisted extraction of phenolic compounds from tiger nut by-products has been established using a Box–Behnken design combined with RSM. The optimal selected conditions were 50 min, 40% ethanol, and ratio solvent/sample 80 *v/w*. The results highlighted that ultrasound technology permits a high recovery of polyphenols from tiger nut by-product. Moreover, PDO and n-PDO tiger nut by-products were compared for the first time according to their phenolic composition and antioxidant activity. Both were characterized by HPLC-MS, and a total of 45 free polar compounds were identified, from which 27 polar compounds were identified for the first time in tiger nut by-products. The predominant compounds were phenolic acids, the major ones being 2-*O*-galloyl-1,4-galactarolactone and vanillic acid derivatives. The quality attributed to the Protected Designation of Origin of Valencia has been confirmed in the PDO tiger nut by-product as it showed higher amounts of phenolic acids and other polyphenols also exhibiting higher antioxidant activity than the n-PDO tiger nut by-product. Further research, with a more robust sampling, could confirm the added value of Protected Designation of Origin of Valencia tiger nut by-products compared to n-PDO options.

To conclude, taking into account that the use of tiger nut flour for gluten-free products was studied, tiger nut by-products could be used as cheaper ingredients/flours for the formulation of bakery products.

## Figures and Tables

**Table 1:** Box–Behnken design with natural and coded values (parenthesis) of the conditions of extraction and the experimental results obtained for total phenolic content (TPC) expressed with the average and the standard deviation.

Run	Independent Factors			Response
	X_1_	X_2_	X_3_	TPC (µg GAE/g d.w.)
1	5 (−1)	0 (−1)	40 (0)	124.02 ± 1.60
2	85 (1)	0 (−1)	40 (0)	142.00 ± 1.46
3	5 (−1)	100 (1)	40 (0)	57.95 ± 0.91
4	85 (1)	100 (1)	40 (0)	93.64 ± 0.40
5	5 (−1)	50 (0)	20 (−1)	135.34 ± 1.44
6	85 (1)	50 (0)	20 (−1)	100.73 ± 1.74
7	5 (−1)	50 (0)	100 (1)	278.72 ± 1.67
8	85 (1)	50 (0)	100 (1)	383.11 ± 1.76
9	45 (0)	0 (−1)	20 (−1)	77.80 ± 1.11
10	45 (0)	100 (1)	20 (−1)	42.80 ± 1.08
11	45 (0)	0 (−1)	100 (1)	281.54 ± 1.48
12	45 (0)	100 (1)	100 (1)	221.53 ± 1.12
13	45 (0)	50 (0)	40 (0)	305.47 ± 1.06
14	45 (0)	50 (0)	40 (0)	291.97 ± 1.93
15	45 (0)	50 (0)	40 (0)	298.69 ± 1.75

X_1–3_: Time (min), ethanol/water (% *v/v*), and solvent/sample ratio (*v/w*).

**Table 2:** Estimated regression effects of the fitted second-order polynomial equation and ANOVA of the fitted model.

Regression Coefficients	Response			
	Effect	Standard Error	*t*-Value	*p*-Value
β_0_ *	178.4055	1.9924	89.5448	0.0001
Lineal				
β_1_ *	46.8713	5.0403	9.2993	0.0114
β_2_ *	−52.3614	4.7817	−10.9505	0.0082
β_3_ *	201.9806	4.7817	42.2407	0.0006
Quadratic				
β_11_ *	62.9065	3.5192	17.8752	0.0031
β_22_ *	131.3867	3.5192	37.3342	0.0007
β_33_ *	61.8885	3.7167	16.6515	0.0036
Crossed				
β_12_	8.8587	6.7623	1.3100	0.3204
β_13_ *	63.7294	6.3755	9.9959	0.0099
β_23_	−12.5084	6.7623	−1.8497	0.2056
R^2^	0.9891			
*p* model	0.0000			
*p* lack of fit	0.2304			

*: Significant at 0.05.

**Table 3:** Polar compounds tentatively identified by HPLC-ESI-TOF-MS.

No.	Compound	Retention Time (min)	Molecular Formula	*m/z* Experimental	*m/z* Calculated	Fragments	Score (%)	Error (ppm)
1	2-*O*-Galloyl-1,4-galactarolactone	0.274	C_13_H_12_O_11_	343.0308	343.0301	201.0249	90.7	2.0
2	Scopoletin	0.520	C_10_H_8_O_4_	191.1686	191.1680	111.0071; 174.0401; 160.8401	100	−3.1
3	Imbricantonol	1.155	C_26_H_20_O_7_	443.1111	443.1131	214.9512; 229.0186; 570.0947	93.9	−4.5
4	*p*-hydroxybenzoic acid	1.341	C_7_H_6_O_3_	137.0234	137.0239	-	100	−3.6
5	l-leucic acid	1.916	C_6_H_12_O_3_	131.0702	131.0708	85.0646	100	−4.6
6	Vanillic acid	2.570	C_8_H_8_O_4_	167.0338	167.0344	-	100	−3.6
7	Ethyl vanillin	3.877	C_9_H_10_O_3_	165.0556	165.0552	151.8809; 136.9292	100	2.4
8	4-vinylphenol	4.643	C_8_H_8_O	119.0492	119.0497	-	100	−4.2
9	Ferulic acid	5.515	C_10_H_10_O_4_	193.0504	193.0501	134.0359; 166.9101	100	1.6
10	*p*-coumaric acid	5.734	C_9_H_8_O_3_	163.0403	163.0395	119.0499; 117.0318; 149.0263	100	4.9
11	3-Hydroxyphloretin 2′-*O*-glucoside (aspalathin)	7.505	C_21_H_24_O_11_	451.3276	451.3271	225.1289; 337.1716; 433.2887; 291.9893; 189.9602;	100	1.1
12	Kaempferol 3,7-diglucoside, sophoraflavonoloside or luteolin-7,3′-di-*O*-glucoside	8.246	C_27_H_30_O_16_	609.1453	609.1456	297.0602; 153.0205; 507.1086; 285.0299; 447.0884	93.4	−0.5
13	Dehydrodivanillin	9.467	C_16_H_14_O_6_	301.0698	301.0712	286.0476; 166.9093; 215.0308; 239.0385	99.9	−4.7
14	Veronicafolin 3-glucosyl-(1->3)-galactoside	9.652	C_30_H_36_O_18_	683.1799	683.1823	721.1418; 593.1249; 563.1607	94.7	−3.5
15	Sinensetin	10.368	C_20_H_20_O_7_	371.1117	371.1131	175.0426; 193.0490; 145.0287; 161.0240; 161.9289	97.6	−3.8
16	Sinapyl alcohol	11.642	C_11_H_14_O_4_	209.0807	209.0814	193.0477; 175.0416	100	−3.3
17	*p*-Coumaric acid ethyl ester	12.672	C_11_H_12_O_3_	191.0699	191.0708	165.0252; 195.0582; 119.0503; 116.9892; 179.0338;	100	−4.7
18	Trihydroxy octadecenoic acid isomer a	14.400	C_18_H_34_O_5_	329.2334	329.2328	229.1451; 211.1347; 171.1032; 183.1397	99.8	1.8
19	Trihydroxy octadecenoic acid isomer b	14.550	C_18_H_34_O_5_	329.2332	329.2328	229.1451; 211.1347; 171.1032; 183.1397	95.3	1.2
20	Trihydroxy octadecenoic acid isomer c	15.295	C_18_H_34_O_5_	329.2325	329.2328	229.1451; 211.1347; 171.1032; 183.1397	99.6	−0.9
21	Cyanidin	15.853	C_15_H_11_O_6_	286.2408	286.2400	265.0123; 116.1101	92.3	0.7
22	Benzoic acid	16.131	C_19_H_20_O_7_	359.1118	359.1131	311.1693; 183.0196; 163.0394; 149.0360	98.4	−3.6
23	Dihydroxyoleic acid acid isomer a	16.487	C_18_H_34_O_4_	313.237	313.2379	183.1375;295.2259; 269.0670	100	−2.9
24	Dihydroxyoleic acid isomer b	16.570	C_18_H_34_O_4_	313.2374	313.2379	201.1127; 223.0940; 171.1013	100	−1.6
25	Dihydroxyoleic acid isomer c	16.830	C_18_H_34_O_4_	313.2367	313.2379	171.1015; 277.2166; 295.2282	100	−3.8
26	Dihydroxyoleic acid isomer d	16.917	C_18_H_34_O_4_	313.2376	313.2379	157.0854; 171.1016; 187.0971	100	−1.0
27	Dihydroxystearic acid	16.975	C_18_H_36_O_4_	315.2531	315.2535	297.2422; 279.2317	100	−1.3
28	Hydroxylinoleic acid isomer a	17.091	C_18_H_32_O_3_	295.2263	295.2273	277.2172; 187.0946; 171.1006	100	−3.4
29	Hydroxypalmitic acid	17.120	C_16_H_32_O_3_	271.2262	271.2273	187.0974; 152.9931; 125.0954	100	−4.1
30	Hydroxylinoleic acid isomer b	17.202	C_18_H_32_O_3_	295.2266	295.2273	277.2162; 171.1014; 195.1375	100	−2.4
31	Hydroxyoleic acid isomer a	17.302	C_18_H_34_O_3_	297.2418	297.2430	279.2306	100	−4.0
32	Hydroxyoleic acid isomer b	17.339	C_18_H_34_O_3_	297.2418	297.2430	279.2319	100	−4.0
33	Hydroxyoleic acid isomer c	17.384	C_18_H_34_O_3_	297.2421	297.2430	279.2316	100	−3.0
34	Hydroxylinoleic acid isomer c	17.442	C_18_H_32_O_3_	295.2263	295.2273	277.2166; 171.1017	100	−3.4
35	Hydroxystearic acid	17.538	C_18_H_36_O_3_	299.2575	299.2586	281.2489; 253.2527	96.7	−3.7
36	Hydroxyoleic acid isomer d	17.621	C_18_H_34_O_3_	297.2422	297.2430	279.2321	100	−2.7
37	Linolenic acid	17.753	C_18_H_30_O_2_	277.2156	277.2168	279.2310; 255.2310	100	−4.3
38	Myristic acid	17.782	C_14_H_28_O_2_	227.2001	227.2011	152.9942; 209.0703	100	−4.4
39	Palmitoleic acid	17.865	C_16_H_30_O_2_	253.2161	253.2168	152.9945	100	−2.8
40	Linoleic acid	17.923	C_18_H_32_O_2_	279.2319	279.2324	152.9952; 255.2314; 241.0059	100	−1.8
41	Methylpalmitic acid	18.009	C_17_H_32_O_2_	267.2319	267.2324	255.2336; 253.2151; 152.9937	96.2	−1.9
42	Palmitic acid	18.088	C_16_H_32_O_2_	255.2316	255.2324	152.9935	99.6	−3.1
43	Oleic acid	18.121	C_18_H_34_O_2_	281.2472	281.2481	255.2308; 152.9945	99.2	−3.2
44	Heptadecanoic acid	18.216	C_17_H_34_O_2_	268.2473	268.2481	269.2477; 255.2326; 152.9957	98.7	−3.0
45	Stearic acid	18.344	C_18_H_36_O_2_	283.2629	283.2637	255.2336; 279.2322; 152.9945	99.3	−2.8

**Table 4:** Phenolic compounds and their precursors quantified in PDO and n-PDO tiger nut by-product expressed as mean ± standard deviation (µg/g dry weight (d.w.)).

No.	Compound	PDO by-Product (µg/g d.w.)	n-PDO by-Product (µg/g d.w.)
1	2-*O*-Galloyl-1,4-galactarolactone	29.17 ± 2.55 ^b^	5.00 ± 0.28 ^a^
2	Scopoletin	3.58 ± 0.05 ^b^	1.44 ± 0.03 ^a^
3	Imbricantonol	0.63 ± 0.04 ^b^	<LOQ ^a^
4	*p*-hydroxybenzoic acid	0.62 ± 0.03 ^b^	<LOQ ^a^
5	Vanillic acid	1.26 ± 0.01 ^b^	0.97 ± 0.02 ^a^
6	Ethyl vanillin	5.60 ± 0.13 ^b^	0.55 ± 0.01 ^a^
7	4-vinylphenol	3.73 ± 0.00 ^b^	0.88 ± 0.02 ^a^
8	Ferulic acid	1.84 ± 0.02 ^b^	1.19 ± 0.06 ^a^
9	*p*-coumaric acid	0.77 ± 0.15 ^b^	0.16 ± 0.08 ^a^
10	3-Hydroxyphloretin 2′-*O*-glucoside (aspalathin)	0.19 ± 0.03 ^a^	0.18 ± 0.00 ^a^
11	Kaempferol 3,7-diglucoside, sophoraflavonoloside or luteolin-7,3′-di-*O*-glucoside	0.14 ± 0.01 ^a^	0.10 ± 0.02 ^a^
12	Dehydrodivanillin	2.27 ± 0.04 ^b^	0.38 ± 0.07 ^a^
13	Veronicafolin 3-glucosyl-(1->3)-galactoside	0.23 ± 0.00 ^a^	0.31 ± 0.01 ^b^
14	Sinensetin	0.03 ± 0.00 ^a^	0.03 ± 0.00 ^a^
15	Sinapyl alcohol	1.37 ± 0.41 ^a^	2.49 ± 0.58 ^a^
16	*p*-Coumaric acid ethyl ester	0.60 ± 0.19 ^a^	<LOQ ^a^
17	Cyanidin	0.67 ± 0.09 ^a^	1.01 ± 0.03 a
18	Benzoic acid	1.37 ± 0.58 ^a^	2.47 ± 0.02 ^a^
	Sum of phenolic compounds	54.07 ± 4.33 ^b^	17.16 ± 1.23 ^a^

Different letters (a, b) in the same line indicate significant differences (*p* < 0.05).

**Table 5:** Antioxidant activity of PDO and n-PDO tiger nut by-product expressed as mean ± standard deviation (µg Trolox equivalent (TE)/g dry weight (d.w.)).

Antioxidant Assay	PDO by-Product (µg/g d.w.)	n-PDO by-Product (µg/g d.w.)
DPPH	434.41 ± 4.73 ^b^	356.90 ± 5.56 ^a^
ABTS	834.61 ± 9.62 ^b^	726.22 ± 7.27 ^a^
FRAP	757.48 ± 6.55 ^b^	618.31 ± 7.89 ^a^

Different letters (a, b) in the same line indicate significant differences (*p* < 0.05).

## Data Availability

Not applicable.
